# Supplementary material for: Non-coding genetic variants underlying higher prostate cancer risk in men of African ancestry
Source: Nat Commun. 2025 Nov 20;16:10202. doi: 10.1038/s41467-025-64631-4 (PMC12635056; doi:10.1038/s41467-025-64631-4)
Supplement: Supplementary file 1 — Supplementary Information [file 41467_2025_64631_MOESM1_ESM.pdf]

# Supplementary Methods

## I. Robustness of prostate enhancer model trained in LNCaP cell line

We applied the LNCaP-trained enhancer model to prioritize non-coding genetic variants. However, to ensure robustness and general validaty of this model, here we ascertained that the enhancer sequence features of LNCaP cells closely resemble those of other prostate cancer cell line (VCaP) and normal primary prostate tissues. We also expanded the training set to include multiple PrCa cell line as well as normal primary prostate tissues to test the robustness of the model.

Toward this, we first evaluated the robustness of the LNCaP model in VCaP cell line. Training our model requires context-specific H3K27ac data. Our analyses suggest that the sequence features of VCaP align well with those of LNCaP to a large extent (Fig S1AB); specifically, the accuracy (auROC) of predicting LNCaP enhancers using VCaP-trained enhancer model is 0.88 (Fig S1A), while the auROC for predicting VCaP enhancers using LNCaP-trained enhancer model is 0.87 (Fig S1B). To further examine whether eSNPs exhibit consistent effect sizes in modulating enhancer activity (measured as average delta across all sliding windows) using different models, we applied the VCaP-trained model to score the 2,000 eSNPs and compared the delta values with those obtained using the LNCaP-trained model. The two resulting average delta scores exhibited a strong correlation with those generated by the LNCaP model, with a Spearman correlation of 0.754 (Fig S1C).

Next, we evaluated the accuracy of the normal primary prostate enhancer model in predicting LNCaP enhancers and vice versa. Additionally, we construct a prostate integrated enhancer model by merging all open chromatin regions (DHS or ATAC-seq) and H3K27ac data from the two prostate cancer cell lines (LNCaP and VCaP) and normal primary prostate tissues, respectively. This model was built using 1KB merged open chromatin regions (DHS or ATAC-seq) that overlap with merged H3K27ac peaks. The goal was to test whether the LNCaP enhancer model could obtain high accuracy across multiple prostate cell lines including the integrated enhancer set. First, using the H3K27ac and ATAC-seq data of normal prostate tissue in Pomerantz et al.[64] to train an enhancer model in normal primary prostate tissues, we observed that the auROC for predicting LNCaP enhancers was 0.72 (Fig S1A). Moreover, the auROC for predicting primary prostate enhancers using the LNCaP-trained model was 0.8 (Fig S1B). More importantly, the LNCaP enhancer model could accurately predict the prostate integrative enhancer regions (auROC = 0.87) (Fig S1B), indicating that the enhancer model based on LNCaP cell line is able to effectively capture prostate-specific sequence features.

In addition, the delta scores of eSNPs derived from LNCaP enhancer model correlates well (Spearman correlations = 0.75, 0.56, 0.68) with those based on other prostate enhancer models (Fig S1C-E).

Therefore, in summary, using the LNCaP model to prioritize SNPs is robust and generally applicable for our purposes.

## II. FOXA1 ChIP-seq experiments in two PrCa cell lines

Per current ENCODE consortium guidelines for transcription factor ChIP-seq, we performed two biological replicates with corresponding input control. Library complexity as determined by non-redundant fraction (NRF) and PCR bottlenecking coefficients 1 and 2 (PBC1, PBC2) passed recommended values. Additional quality assessment metrics such as fraction of reads in peaks (FRiP score) are provided in Table below. Notably the most enriched motif in significant peaks is FOXA1 with p-value an order of magnitude greater than next enriched motif, consistent with specificity of ChIP-seq for FOXA1.

## Table S1. LNCaP Replicate1:

| Rank | Motif | P-value | log P-pvalue | % of Targets | % of Background | STD(Bg STD) | Best Match/Details |
|---|---|---|---|---|---|---|---|
| 1 | 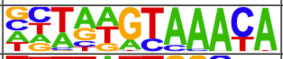 | 1e-11314 | -2.605e+04 | 59.77% | 16.80% | 206.0bp (123.6bp) | FOXA1(Forkhead)/LNCAP-FOXA1-ChIP-Seq(GSE27824)/Homer(0.980) More Information \| Similar Motifs Found |
| 2 | 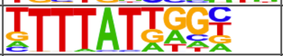 | 1e-1275 | -2.938e+03 | 30.21% | 17.02% | 226.4bp (119.3bp) | Hoxd13(Homeobox)/ChickenMSG-Hoxd13.Flag-ChIP-Seq(GSE86088)/Homer(0.968) More Information \| Similar Motifs Found |
| 3 | 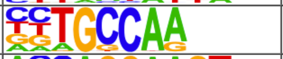 | 1e-970 | -2.235e+03 | 38.91% | 25.97% | 207.3bp (122.5bp) | NFIX/MA0671.1/Jaspar(0.972) More Information \| Similar Motifs Found |
| 4 | 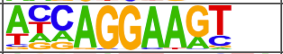 | 1e-306 | -7.049e+02 | 9.00% | 5.15% | 220.2bp (115.2bp) | ELF3(ETS)/PDAC-ELF3-ChIP-Seq(GSE64557)/Homer(0.987) More Information \| Similar Motifs Found |
| 5 | 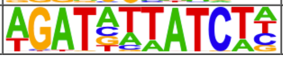 | 1e-293 | -6.767e+02 | 4.10% | 1.72% | 214.0bp (115.0bp) | GATA(Zf),IR3/iTreg-Gata3-ChIP-Seq(GSE20898)/Homer(0.957) More Information \| Similar Motifs Found |

## Table S2. LNCaP Replicate 2:

| Rank | Motif | P-value | log P-pvalue | % of Targets | % of Background | STD(Bg STD) | Best Match/Details |
|---|---|---|---|---|---|---|---|
| 1 | 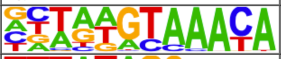 | 1e-13058 | -3.007e+04 | 59.68% | 16.60% | 194.8bp (113.2bp) | FOXA1(Forkhead)/LNCAP-FOXA1-ChIP-Seq(GSE27824)/Homer(0.982) More Information \| Similar Motifs Found |
| 2 | 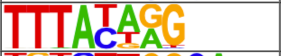 | 1e-1289 | -2.970e+03 | 27.49% | 15.51% | 211.7bp (111.3bp) | Hoxa13(Homeobox)/ChickenMSG-Hoxa13.Flag-ChIP-Seq(GSE86088)/Homer(0.900) More Information \| Similar Motifs Found |
| 3 | 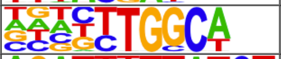 | 1e-1082 | -2.492e+03 | 32.69% | 20.72% | 200.3bp (113.3bp) | NFIX/MA0671.1/Jaspar(0.976) More Information \| Similar Motifs Found |
| 4 | 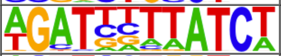 | 1e-345 | -7.951e+02 | 4.16% | 1.74% | 215.7bp (103.7bp) | GATA(Zf),IR4/iTreg-Gata3-ChIP-Seq(GSE20898)/Homer(0.941) More Information \| Similar Motifs Found |
| 5 | 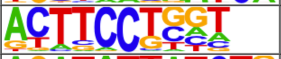 | 1e-338 | -7.800e+02 | 12.86% | 8.26% | 209.0bp (108.6bp) | EHF(ETS)/LoVo-EHF-ChIP-Seq(GSE49402)/Homer(0.975) More Information \| Similar Motifs Found |
| 6 | 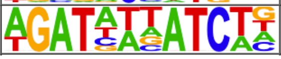 | 1e-264 | -6.084e+02 | 3.10% | 1.28% | 204.9bp (106.6bp) | GATA(Zf),IR3/iTreg-Gata3-ChIP-Seq(GSE20898)/Homer(0.979) More Information \| Similar Motifs Found |

## Table S3. MDA_PCa2b Replicate 1:

| Rank | Motif | P-value | log P-pvalue | % of Targets | % of Background | STD(Bg STD) | Best Match/Details |
|---|---|---|---|---|---|---|---|
| 1 | 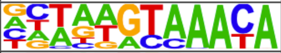 | 1e-8987 | -2.069e+04 | 59.99% | 15.12% | 170.3bp (115.5bp) | FOXA1(Forkhead)/LNCAP-FOXA1-ChIP-Seq(GSE27824)/Homer(0.980) More Information \| Similar Motifs Found |
| 2 | 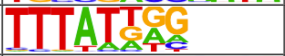 | 1e-1099 | -2.532e+03 | 36.78% | 21.11% | 186.9bp (117.0bp) | HOXB13(Homeobox)/ProstateTumor-HOXB13-ChIP-Seq(GSE56288)/Homer(0.934) More Information \| Similar Motifs Found |
| 3 | 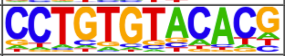 | 1e-742 | -1.709e+03 | 40.41% | 26.81% | 181.5bp (119.1bp) | ZSCAN29/MA1602.1/Jaspar(0.737) More Information \| Similar Motifs Found |
| 4 | 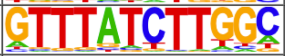 | 1e-485 | -1.118e+03 | 9.62% | 4.11% | 182.1bp (113.7bp) | NF1:FOXA1(CTF,Forkhead)/LNCAP-FOXA1-ChIP-Seq(GSE27824)/Homer(0.829) More Information \| Similar Motifs Found |
| 5 | 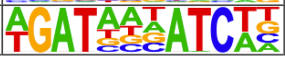 | 1e-349 | -8.038e+02 | 5.70% | 2.17% | 183.5bp (108.3bp) | GATA(Zf),IR3/iTreg-Gata3-ChIP-Seq(GSE20898)/Homer(0.980) More Information \| Similar Motifs Found |

## Table S4. MDA_PCa2b Replicate 2:

| Rank | Motif | P-value | log P-pvalue | % of Targets | % of Background | STD(Bg STD) | Best Match/Details |
|---|---|---|---|---|---|---|---|
| 1 | 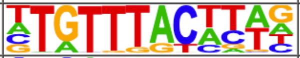 | 1e-13815 | -3.181e+04 | 64.21% | 21.01% | 199.8bp (113.9bp) | FOXA1(Forkhead)/LNCAP-FOXA1-ChIP-Seq(GSE27824)/Homer(0.991) More Information \| Similar Motifs Found |
| 2 | 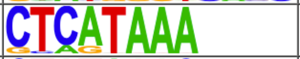 | 1e-1255 | -2.891e+03 | 23.63% | 13.25% | 216.8bp (110.8bp) | PH0068.1_Hoxc13/Jaspar(0.901) More Information \| Similar Motifs Found |
| 3 | 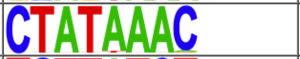 | 1e-622 | -1.433e+03 | 17.71% | 11.05% | 218.6bp (112.2bp) | POL012.1_TATA-Box/Jaspar(0.767) More Information \| Similar Motifs Found |
| 4 | 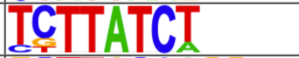 | 1e-608 | -1.401e+03 | 30.02% | 21.65% | 223.1bp (110.0bp) | GATA6/MA1104.2/Jaspar(0.976) More Information \| Similar Motifs Found |
| 5 | 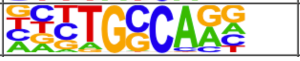 | 1e-528 | -1.216e+03 | 24.65% | 17.42% | 209.9bp (113.9bp) | NF1-halfsite(CTF)/LNCaP-NF1-ChIP-Seq(Unpublished)/Homer(0.977) More Information \| Similar Motifs Found |

For Figure 5D, we used bamCoverage from deeptools/3.5.1 to normalize for read depth using reads per genomic content (RPGC) with a bin size of 25 bp and effective genome size of 2.7 billion. We then took the average of the normalized signals of duplicates of each cell line for comparison of signal intensities across the two cell lines.

In addition, below are examples of QC metrics for those ChIP experiments. Note the top scoring peak is forkhead with highly significant p-value.

| SampleName | Unique_Mapped | Dup_Rate | NRF | PBC1 | PBC2 | FRiP | Peaks | Peaks>10 | Motif | p_value |
|---|---|---|---|---|---|---|---|---|---|---|
| KF3_lncap_foxa1_S2 | 86.18 | 0.07 | 0.915 | 0.928 | 14.08 | 0.16144 | 72502 | 27279 | FOXA1 | 1e-11314 |
| KF6_lncap_foxa1_kf_S7 | 79.15 | 0.09 | 0.912 | 0.914 | 11.735 | 0.18293 | 83948 | 34488 | FOXA1 | 1e-13058 |
| KF3_lncap_input_S1 | 54.78 | 0.06 | 0.933 | 0.94 | 16.854 | NA | NA | NA | NA | NA |
| KF6_lncap_input_S5 | 98.15 | 0.07 | 0.929 | 0.934 | 15.274 | NA | NA | NA | NA | NA |
| KF3_mda_foxa1_S4 | 52.48 | 0.06 | 0.932 | 0.937 | 15.876 | 0.11506 | 52048 | 21465 | FOXA1 | 1e-8987 |
| KF6_mda_foxa1_kf_S10 | 57.27 | 0.09 | 0.91 | 0.911 | 11.245 | 0.23992 | 98662 | 42886 | FOXA1 | 1e-13815 |
| KF3_mda_input_S3 | 51.7 | 0.06 | 0.929 | 0.937 | 16.023 | NA | NA | NA | NA | NA |
| KF6_mda_input_S8 | 54.49 | 0.07 | 0.932 | 0.934 | 15.281 | NA | NA | NA | NA | NA |

QC metrics description

| | |
|---|---|
| Unique_Mapped | Uniquely mapped reads (millions) |
| Dup_Rate | Duplication Rate |
| NRF | Non redundant fraction (> 0.9 is ideal according to ENCODE) |
| PBC1 | PCR bottlenecking coefficient 1 (> 0.9 is none according to ENCODE standards) |
| PBC2 | PCR bottlenecking coefficient 2 (> 3 is none according to ENCODE standards) |
| FRiP | Fraction of reads in peaks |
| Peaks | Number of peaks called by MACS |
| Peaks > 10 | Number of peaks called by MACS with fold change > 10 |
| Motif | Top scoring motif by Homer |
| p_value | p-value of top scoring hit by Homer |

## Supplementary Figures

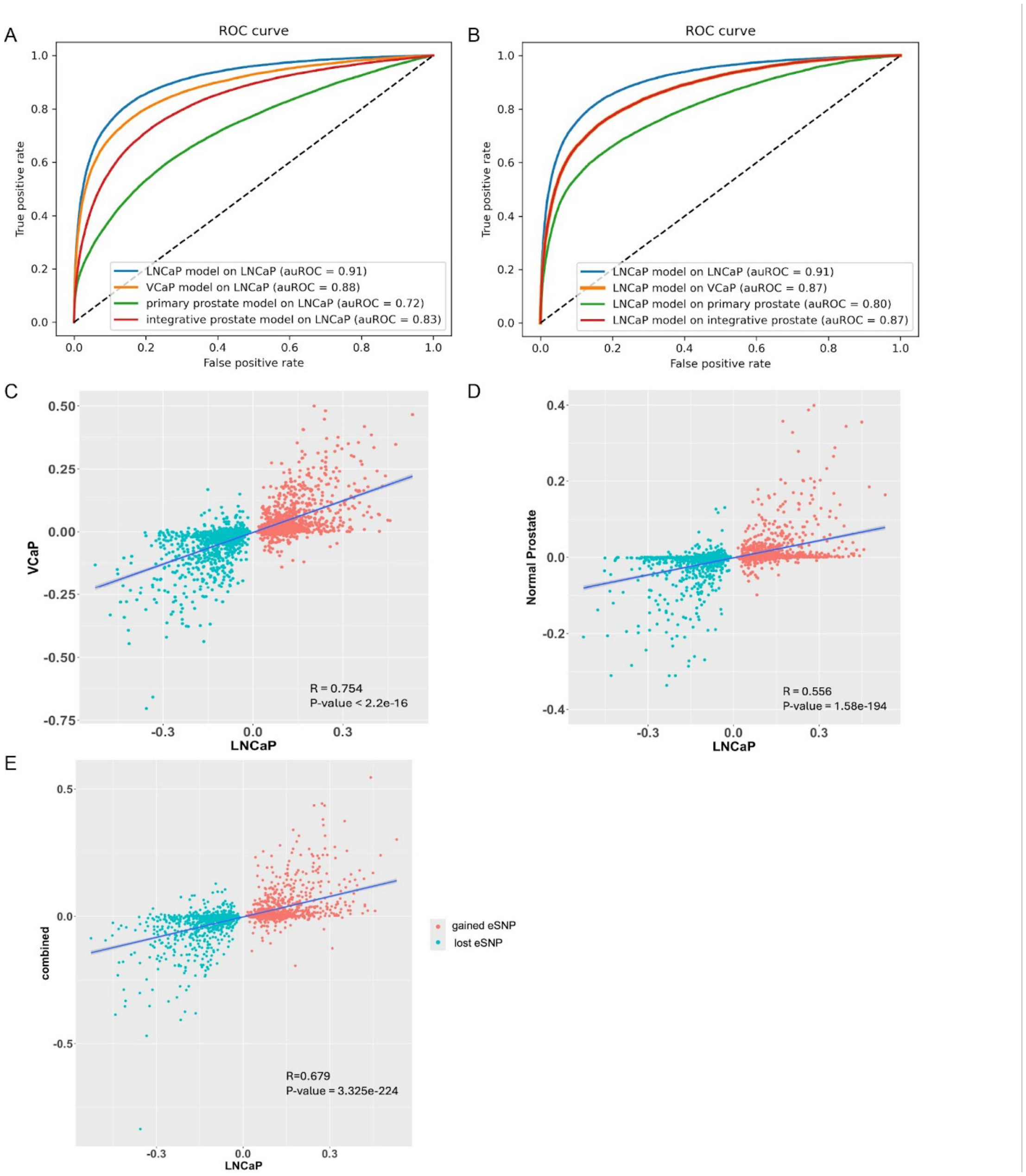


Fig. S1. **Robustness of enhancer model.** A) auROC of models trained on VCaP, normal primary prostate enhancer, or prostate integrated enhancers (enhancers merged from LNCaP, VCaP, and primary prostate) models on LNCaP enhancers. B) auROC of LNCaP-trained enhancer models on VCaP, primary prostate enhancers, and prostate integrated enhancers. C) scatter plot of delta scores of eSNPs using VCaP-trained enhancer model against those using LNCaP-trained enhancer model. D) scatter plot of delta scores of eSNPs using normal prostate enhancer-trained model against those based on LNCaP-trained enhancer model. E) scatter plot of delta scores of eSNPs using prostate integrated enhancers-trained model against those using LNCaP-trained enhancer model. Source data are provided as a Source Data file.

A

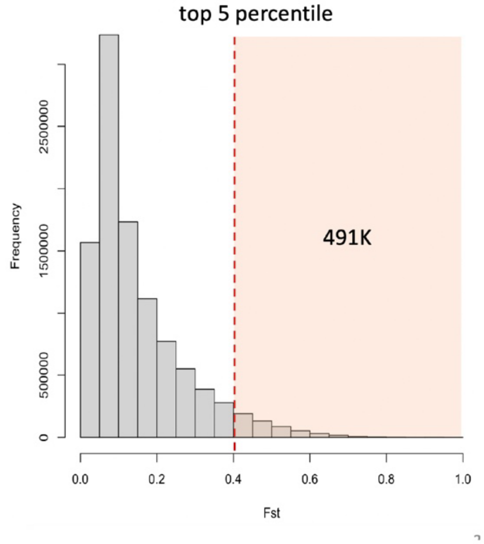


B

| | AA dominant | EA dominant |
|---|---|---|
| gained | 1296 | 773 |
| lost | 1111 | 1064 |

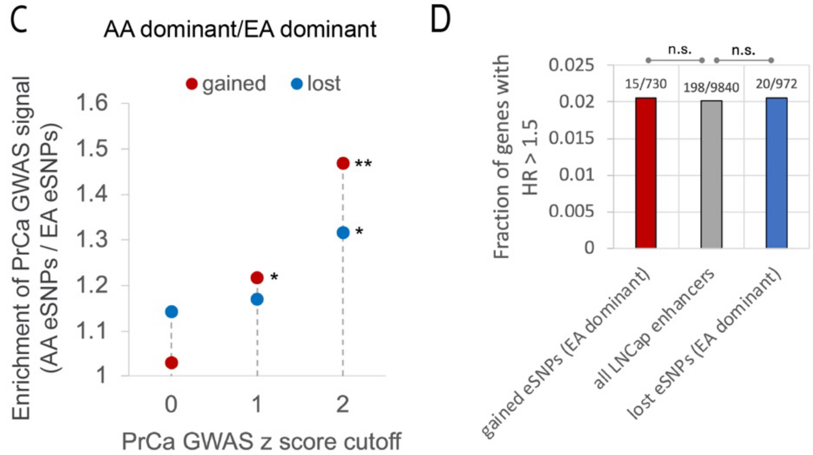


**Fig. S2**. **eSNPs with AA dominant alternate alleles are associated with PrCa risk.** A) Distribution of $\mathbf{F_{ST}}$ of SNPs that are common (MAF ≥ 0.05) in either EA or AA. The shaded part is the top 5% of SNPs with the highest $\mathbf{F_{ST}}$. B) Number of gained and lost eSNPs that are dominant either in AA or EA. C) Enrichment of both gained and lost eSNPs with AA-dominant alleles in PrCa GWAS traits compared to gained and lost eSNPs with EA-dominant alleles, respectively. For gained and lost eSNPs the y-axis shows the ratio of the fraction of AA-dominant alleles with PrCa GWAS signal above the cutoff (x-axis) to the fraction of EA-dominant alleles with PrCa GWAS signal above the cutoff. * indicates p-values ≤ 0.05, and ** indicates p-values ≤ 0.01. The p-values are Bonferroni-corrected and obtained from one-sided Fisher's exact test. D) In the three categories -- EA-dominant gained activity eSNPs, lost activity eSNPs, and all LNCaP enhancers, the figure shows the fraction of genes with HR > 1.5 (Bonferroni-corrected P-value ≤ 0.05). Each pair of categories was compared using one-sided Fisher's exact test. n.s. refers to non-significant based on one-sided Fisher's exact test. Source data are provided as a Source Data file.

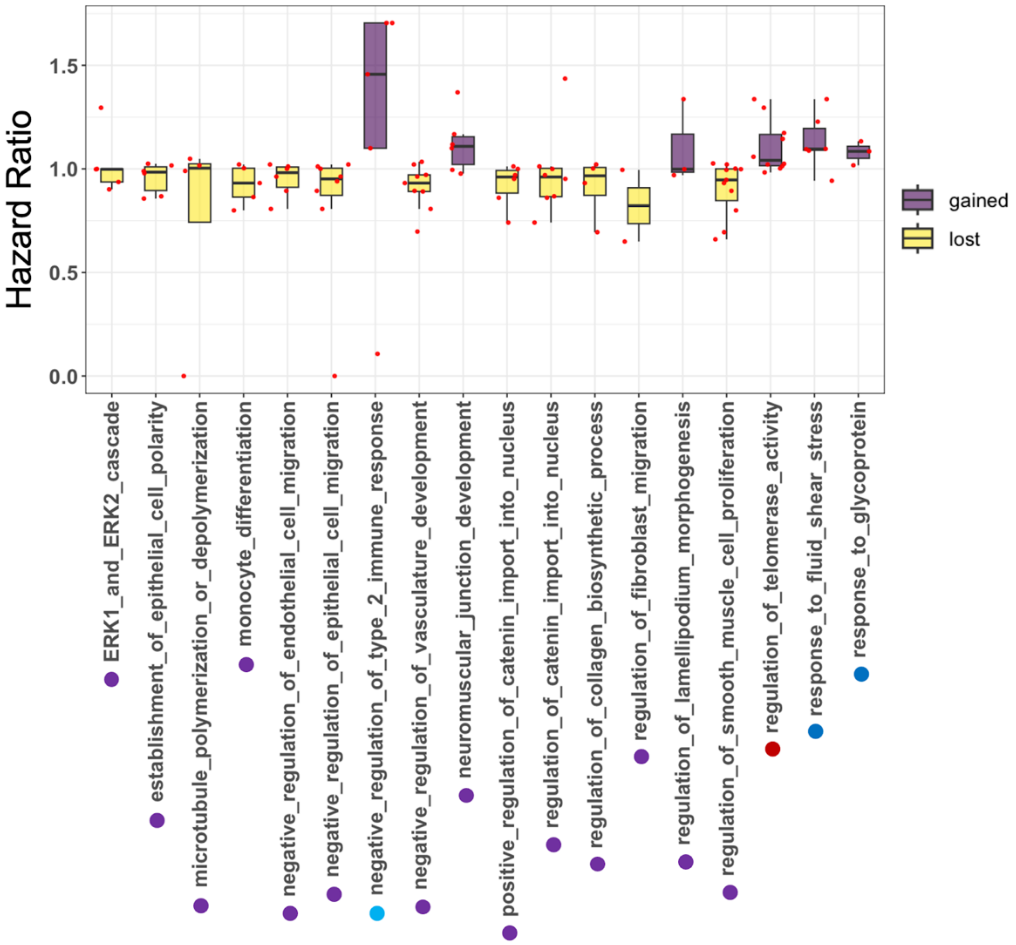


Fig. S3. Hazard Ratios of putative gene targets of gained and lost eSNPs corresponding to the enriched GO terms. Only genes with significant HRs (Bonferroni-corrected P-value ≤ 0.05) were included in the plot. Along x-axis, purple dots are GO terms associated with differentiation/development, light blue dots are associated with regulation of the immune system, dark blue dots are associated with immune response, and red dots are GO terms associated with telomere length regulation. In the boxplot, the horizonal line in the middle is the median value and the lower and upper edges of the boxes correspond to the 25th and 75th percentiles. Extending vertically upwards/downwards of the boxes are the lines showing 1.5 times the interquartile range (i.e., distance between 25th and 75th percentile). From left to right, the number of data points in the boxplots are: n = 5, 6, 4, 5, 6, 8, 5, 9, 6, 6, 8, 4, 2, 3, 11, 10, 6, 3. Source data are provided as a Source Data file.

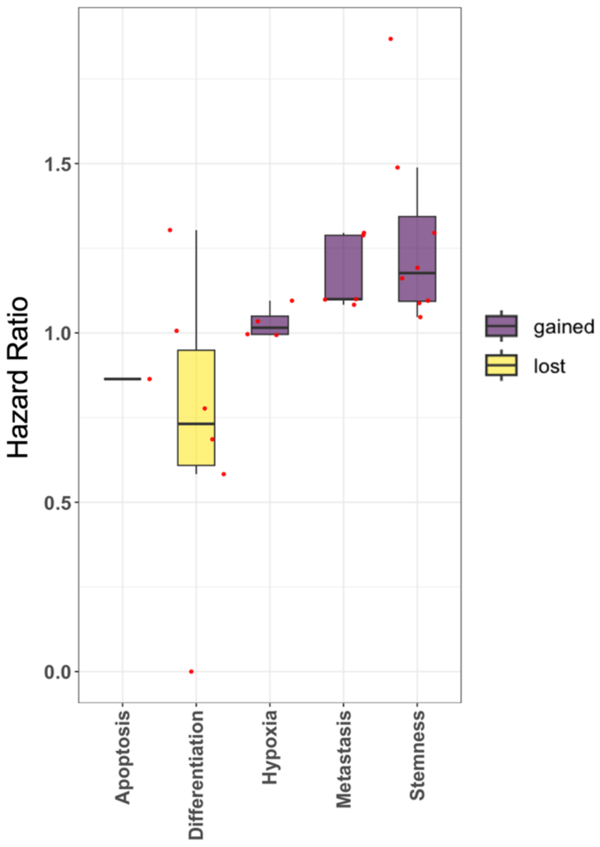


Fig. S4. Hazard Ratios of putative gene targets of gained and lost eSNPs belonging to enriched CancerSEA signatures. Only genes with significant HRs (Bonferroni-corrected one-sided Fisher's exact test P-value ≤ 0.05) were included in the plot. In the boxplot, the horizonal line in the middle is the median value and the lower and upper edges of the boxes correspond to the 25th and 75th percentiles. Extending vertically upwards/downwards of the boxes are the lines showing 1.5 times the interquartile range (i.e., distance between 25th and 75th percentile). From left to right, the number of data points in the boxplots are: n = 1, 6, 4, 5, 8. Source data are provided as a Source Data file.

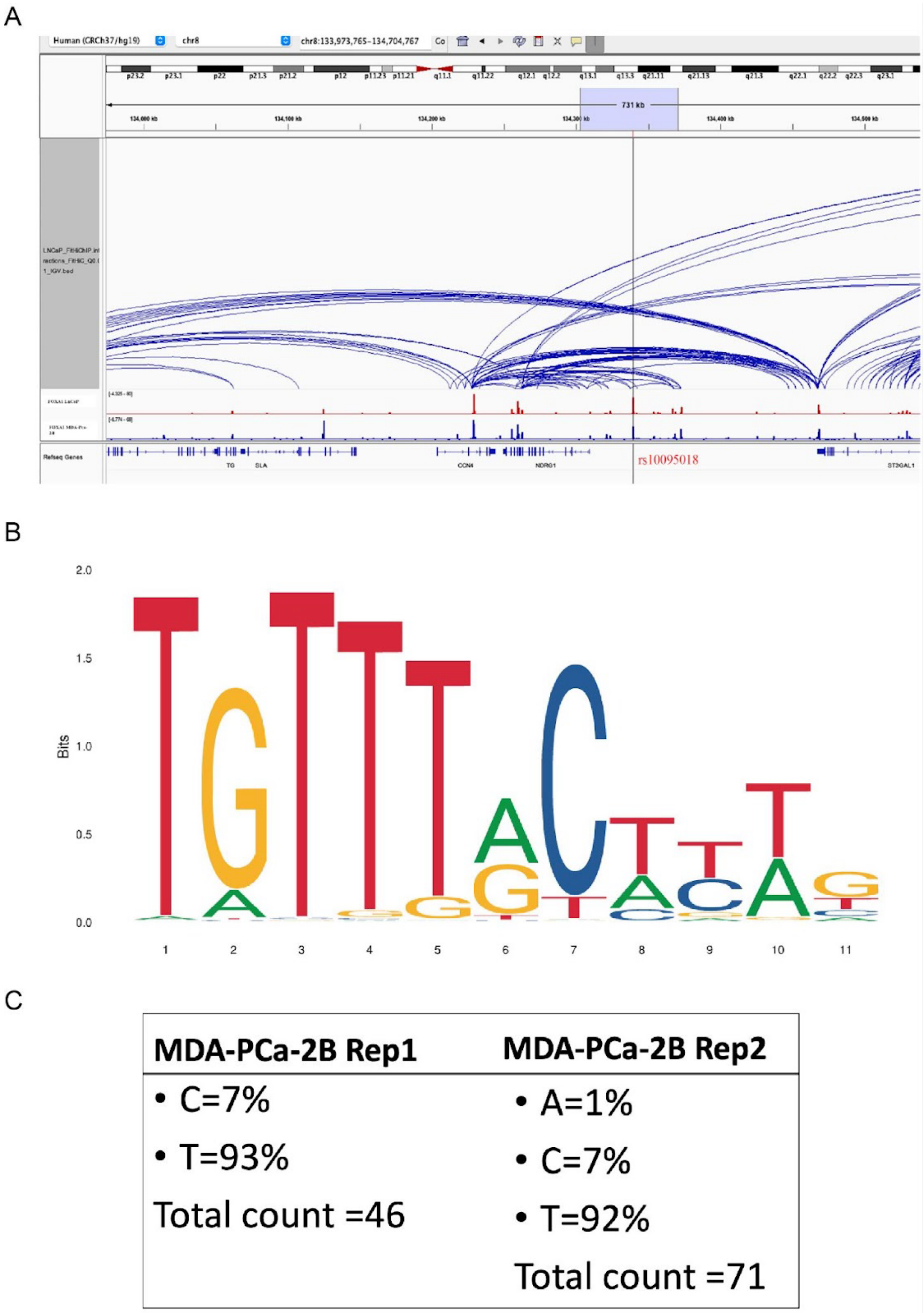


**Fig. S5. An example of lost eSNP rs10095018.** A) LNCaP H3K27ac HiChIP data near the rs10095018 SNP locus. B) An enlarged binding motif logo of FOXA1 (MA0148.1) from JASPAR[56], where the 6th position corresponds to the eSNP. C). Percentage of FOXA1 ChIP-seq reads coverage of both T and C alleles at the rs10095018 SNP site in MDA PCa 2B cell line. Source data are provided as a Source Data file.

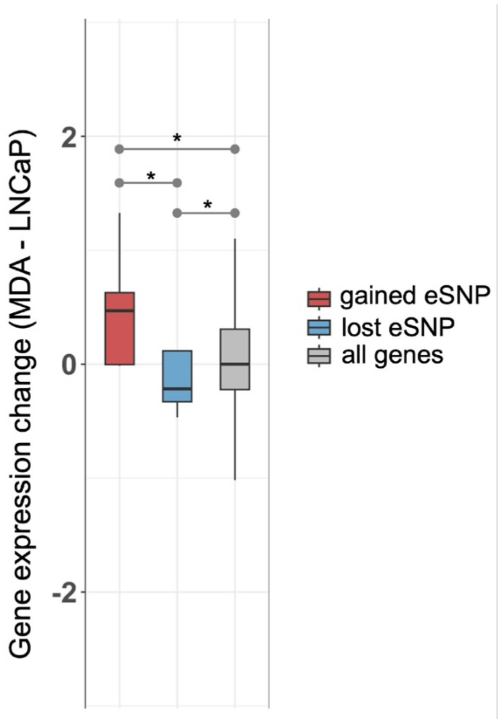


Fig. S6. Putative target (linked by HiChIP loops) gene expression change (MDA - LNCaP) of the genes in 3D contact with gained (n = 12) and lost (n = 10) activity eSNP sites where MDA PCa 2B (dubbed MDA) genotype has a greater number of alternate alleles. All genes (n = 19082) associated with non-eSNP are used as background. In the boxplot, the horizonal line in the middle is the median value and the lower and upper edges of the boxes correspond to the 25th and 75th percentiles. Extending vertically upwards/downwards of the boxes are the lines showing 1.5 times the interquartile range (i.e., distance between 25th and 75th percentile). * indicates Bonferroni-corrected one-sided Wilcoxon P-values < 0.05. Source data are provided as a Source Data file.

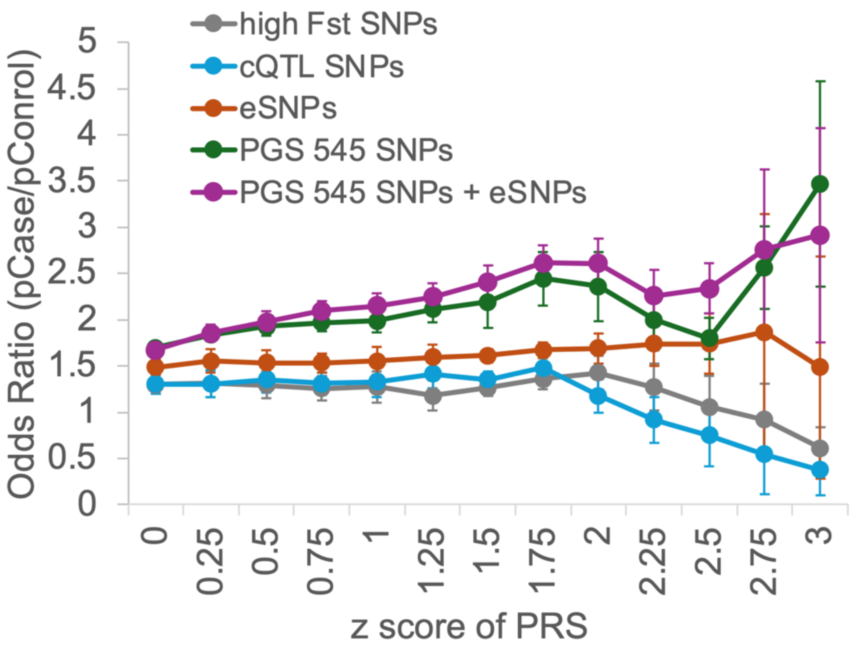


**Fig. S7**. Odds ratio (y-axis) related to different SNP sets based on contrast PRS against PRS score threshold (x-axis) in EA cohort. The odds ratio is the ratio of the fraction of PrCa patients with PRS above a certain threshold to that of control individuals with PRS above the same threshold. All PRSs based on different SNP sets are z-score normalized for comparison. Central dots are the median values. Error bars show the standard deviation of the 10-fold cross validation. Source data are provided as a Source Data file.

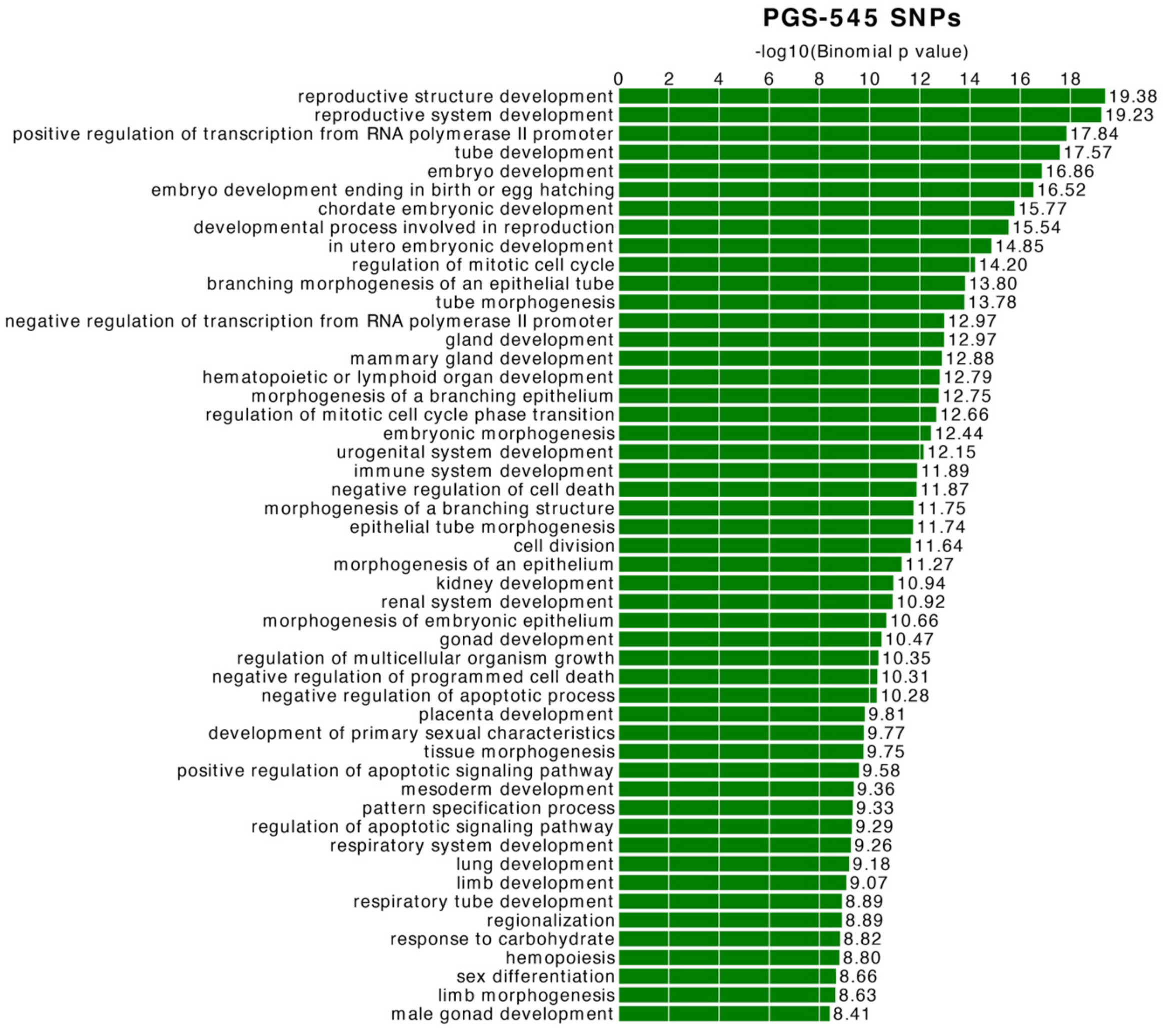


**Fig. S8**. Biological processes associated with gained activity PGS-545 SNPs using the GREAT tool. The bar values are the -log10 of the P-values based on one-sided Binomial test without multiple test adjustment. Source data are provided as a Source Data file.
