# Supplementary material for: Non-coding genetic variants underlying higher prostate cancer risk in men of African ancestry
Source: Nat Commun. 2025 Nov 20;16:10202. doi: 10.1038/s41467-025-64631-4 (PMC12635056; doi:10.1038/s41467-025-64631-4)
Supplement: Supplementary file 3 — Description of Additional Supplementary Files [file 41467_2025_64631_MOESM3_ESM.pdf]

Description of Additional Supplementary Files

Supplementary Data 1. Genes of each GO terms associated with gained and lost eSNPs.

Supplementary Data 2. Hazard Ratio of genes of GO terms associated with eSNPs.

Supplementary Data 3. Transcription factor binding sites enriched at gained eSNP sites.

Supplementary Data 4. List of gained eSNPs.

Supplementary Data 5. Transcription factor binding sites enriched at lost eSNP sites.

Supplementary Data 6. List of lost eSNPs.

Supplementary Data 7. Genes of each CancerSEA signature associated with gained and lost eSNPs related to disturbing FOXA1 binding.

Supplementary Data 8. Hazard Ratio of genes of each cancerSEA signaures.

Supplementary Data 9. Number of eSNPs in each cell line that have larger number of alternative alleles than the other cell line.
